# No Evidence to Support a Causal Relationship between Circulating Adiponectin Levels and Ankylosing Spondylitis: A Bidirectional Two-Sample Mendelian Randomization Study

**DOI:** 10.3390/genes13122270

**Published:** 2022-12-02

**Authors:** Jiale Xie, Mingyi Yang, Hui Yu, Ke Xu, Xianjie Wan, Jiachen Wang, Guoqiang Wang, Peng Xu

**Affiliations:** Department of Joint Surgery, HongHui Hospital, Xi’an Jiaotong University, Xi’an 710054, China

**Keywords:** ankylosing spondylitis, circulating adiponectin levels, single-nucleotide polymorphisms, causal, Mendelian randomization

## Abstract

Based on previous observational studies, the causal association between circulating adiponectin (CA) levels and ankylosing spondylitis (AS) risk remains unclear. Therefore, this study aims to investigate whether CA levels are related to the risk of AS. We carried out a bidirectional two-sample Mendelian randomization (MR) analysis to examine the causal correlation between CA levels and AS via published genome-wide association study (GWAS) datasets. Single-nucleotide polymorphisms (SNPs) related to CA levels were derived from a large GWAS that included 39,883 individuals of European descent. SNPs related to AS were obtained from the FinnGen consortium (2252 cases and 227,338 controls). The random-effects inverse variance weighted (IVW) method was the primary method utilized in our research. We also used four complementary approaches to improve the dependability of this study (MR–Egger regression, Weighted median, Weighted mode, and Simple mode). Random-effects IVW (odds ratio [OR], 1.00; 95% confidence interval [CI], 0.79–1.27, *p* = 0.984) and four complementary methods all indicated that genetically predicted CA levels were not causally related to the risk of AS. In reverse MR analysis, there is little evidence to support the genetic causality between the risk of AS and CA levels.

## 1. Introduction

As a common chronic inflammatory disease, ankylosing spondylitis (AS) mainly manifests as the inflammatory alteration of the spine and the sacroiliac joints that ultimately result in ankylosis and even physical disability [1,2]. It is part of the spectrum of spondyloarthritis (SpA) and affects especially young male individuals aged around 25 years [3,4]. AS is one of the most common forms of arthritis in developed and developing countries [5]. Its global prevalence is approximately 0.1–0.3% in the general population [6]. AS causes inflammatory back pain and brings about an impaired quality of life as well as a huge social burden on patients [7,8]. However, to date, there is no effective way to cure AS, and symptoms can only be relieved by pharmacotherapy [9]. The etiology of AS is complex; however, it is generally believed that genetic, immune, microbial, and endocrine factors are related to its occurrence and progression, of which genetic factors with >90% heritability are the primary cause [2,10]. Even so, the specific pathophysiological process of AS is still incompletely understood. Therefore, further research is necessary on its etiology.

Adiponectin is the most abundant adipokine secreted by adipocytes in human plasma [11]. It participates in multiple physiological activities, such as enhancing insulin sensitivity and promoting anti-inflammatory/antifibrotic abilities [12]. Of note, recent studies have suggested that adiponectin has proinflammatory effects in patients with autoimmune diseases [13,14,15]. The evidence on the relationship between circulating adiponectin (CA) levels and AS is currently inconclusive. A study has shown that reduced high-molecular-weight adiponectin levels are associated with new bone formation in AS [16]. Others found significantly elevated serum adiponectin levels in patients with AS compared with healthy individuals [17]. However, previous meta-analyses have indicated that CA levels were not significantly different between patients with AS and controls [18,19]. The causal relevance between CA levels and AS risk has not yet been established. Therefore, exploring the causality between the two has crucial implications.

Mendelian randomization (MR) is a novel research method that uses single-nucleotide polymorphisms (SNPs) as instrumental variables (IVs) to deduce causal correlations between exposures and outcomes [20]. As genetic variations (SNPs) occur at conception and are almost unaffected by the outcome, they are not subject to confounders or reverse causality [21]. Up to now, MR approaches have been successfully adapted to assess the causal associations of CA levels with osteoarthritis [22], gout [23], and asthma [24].

Herein, we utilized a two-sample MR analysis to examine whether there is a causal connection between CA levels and the risk of AS based on two diverse genome-wide association study (GWAS) databases.

## 2. Materials and Methods

### 2.1. Ethical Considerations

As all the data in our study were obtained from publicly available databases, further ethical approval was not required.

### 2.2. Study Design

**Figure 1:**
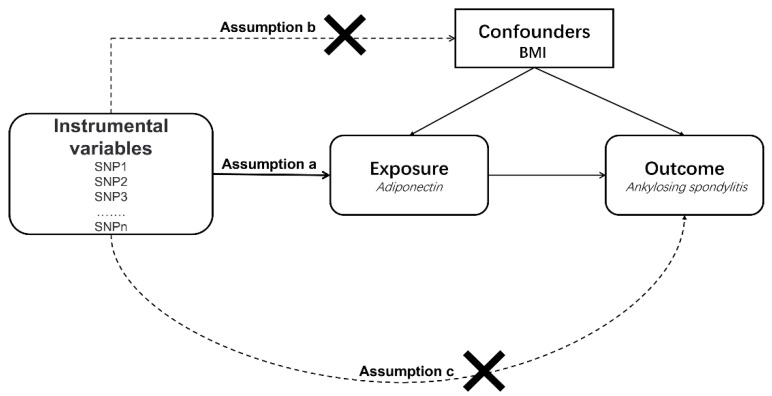
An overview of the study design. SNP, single-nucleotide polymorphism; BMI, body mass index.

In our MR study, SNPs were used as IVs to assess causal effects. The graphical flow of the experimental design is demonstrated in Figure 1. The MR analysis was required to satisfy the following three assumptions:

(a) All selected IVs should be strongly related to exposure;
(b) All selected IVs should not be associated with confounding factors;
(c) All selected IVs only influence the outcome through exposure.

### 2.3. Data Sources

We selected SNPs as IVs for exposure (adiponectin) from the IEU OpenGWAS database (GWAS ID: ieu-a-1, https://gwas.mrcieu.ac.uk/ (accessed on 10 May 2020)), which contains a full summary statistic for over 10,000 GWASs in variant call format [25]. The summarized GWAS data for CA levels were derived from a large-scale GWAS that included 39,883 individuals of European ancestry. In this GWAS study, CA levels were accessed by an enzyme-linked immunosorbent assay or radioimmunity assay. All participants were genotyped via commercially available Affymetrix or Illumina genome-wide genotyping arrays. More details are presented in the published study [26]. The FinnGen consortium (http://www.r7.finngen.fi/ (accessed on 9 June 2022)) provided the GWAS data for outcome (AS), which included 2252 patients with AS and 227,338 controls of Finnish ancestry until 1 June 2022 (FREEZE 7). The current data freeze (DF7) comprises > 309,000 individuals, almost 17 M variants, and 3095 diseases. Additionally, all cases of DF7 were defined by the code M13 in the International Classification of Diseases, Tenth Revision (ICD-10).

### 2.4. Selection of IVs

To obtain SNPs significantly associated with adiponectin, we set *p* < 5 × 10^−8^ as the genome-wide significance threshold. Meanwhile, to eliminate bias due to linkage disequilibrium (LD), the LD of these SNPs that are significantly related to adiponectin levels should meet the circumstances of r^2^ > 0.001 and kb > 10,000. Furthermore, we manually eliminated SNPs related to confounders using PhenoScanner (http://www.Phenoscaner.medschl.cam.ac.uk (accessed on 1 November 2018)). Palindromic SNPs with intermediate allele frequencies were excluded from our MR analyses. We additionally calculated the F-statistics to avoid the bias triggered by weak IVs. An F-statistic of >10 indicated that they were not vulnerable IVs [27]. The F-statistics were calculated by the following formula: F = R^2^ (N − 2)/(1 − R^2^), and R^2^ was calculated by the following formula: R^2^ = 2 × MAF × (1 − MAF) β^2^. MAF, β, and N refer to the minor allele frequency, estimated adiponectin effect, and sample size, respectively [28].

### 2.5. Statistical Analyses

In our MR analysis, we applied multiple approaches to evaluate the causal impact of exposure on the outcome, including the random-effects IVW, MR–Egger regression, Weighted median, Weighted mode, and Simple mode methods. The dominant method for analysis was the random-effects IVW, which provided the most accurate results when all selected IVs were valid [29]. Additionally, we carried out a sequence of sensitivity analyses. The Weighted median method yielded consistent estimates of causal effects when approximately half of the SNPs were invalid IVs [30]. The IVW approach and MR–Egger regression were used to investigate the presence of heterogeneity in our results, which was quantified using Cochran’s Q test [31]. MR–Egger regression was also applied to determine the possibility of pleiotropy, whose intercept term represented the potential horizontal pleiotropy [32]. Furthermore, we used the leave-one-out analysis to investigate the effect of a specific single SNP on the causal relevance between CA levels and the risk of AS [33]. All MR analyses were performed by the package “TwoSampleMR” in R software (version 4.2.1).

## 3. Results

A bidirectional, two-sample MR analysis was conducted to explore the causal connection between CA levels and the risk of AS. Our MR results did not reveal a causal connection between CA levels and AS risk, and we were unable to determine the causal relevance of AS risk on CA levels. 

### 3.1. MR

After setting a genome-wide significance threshold of *p* < 5 × 10^−8^ and removing LD, a total of 14 SNPs were screened. However, two SNPs (rs1108842 and rs731839) were manually removed because they were associated with body mass index, which is considered a major confounder [34]. Finally, we performed MR analysis using 12 SNPs identified as IVs of adiponectin, and more detailed information on the 12 SNPs is shown in Table 1. The F-statistics of these SNPs ranged from 13 to 158, indicating that they were all robust IVs.

**Figure 2:**
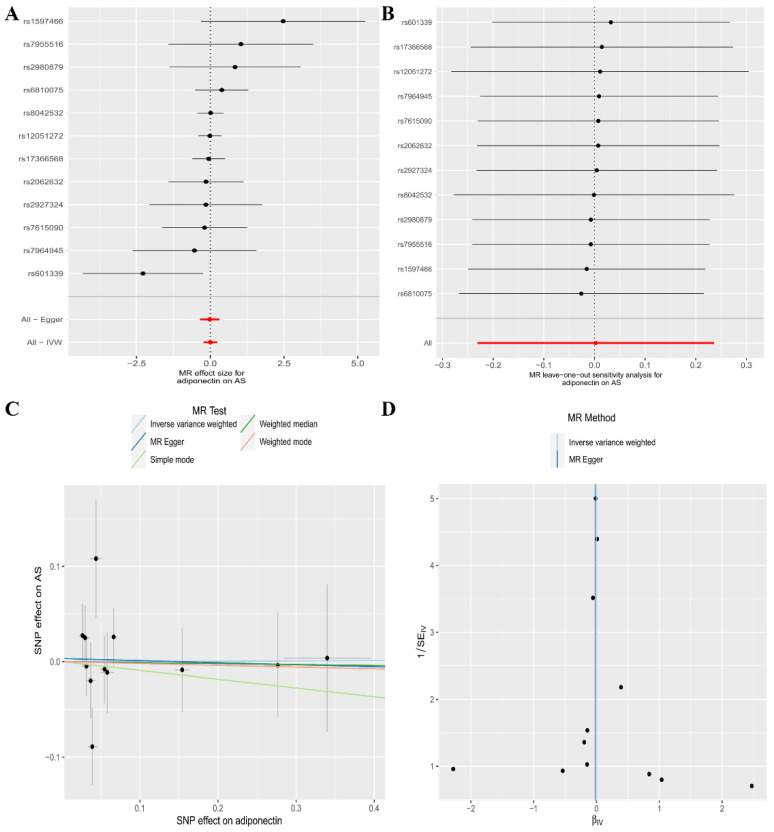
The association between circulating adiponectin levels and the risk of ankylosing spondylitis is presented in (**A**) a forest plot, (**B**) a leave-one-out sensitivity analysis, (**C**) a scatter plot, and (**D**) a funnel plot.

In the current analysis, the IVW (as the primary approach to analysis) approach did not show a causal connection between genetically predicted CA levels and the risk of AS (odds ratio [OR], 1.00; 95% confidence interval [CI], 0.79–1.27, *p* = 0.984). Secondary analysis methods, including the MR–Egger regression (OR, 0.98; 95% CI, 0.70–1.36, *p* = 0.901), Weighted median (OR, 0.99; 95% CI, 0.75–1.31, *p* = 0.945), Weighted mode (OR, 0.98; 95% CI, 0.75–1.28, *p* = 0.890), and Simple mode (OR, 0.91; 95% CI, 0.60–1.38, *p* = 0.653) approaches, all exhibited the same results (Table 2, Figure 2A). The Leave-one-out sensitivity analysis indicated that the absence of a single SNP disproportionately affected the causality estimates of CA levels for the risk of AS (Figure 2B). The MR–Egger regression method confirmed that our results were not affected by horizontal pleiotropy (intercept = 0.003, *p* = 0.843) (Table 2, Figure 2C). The Cochran’s Q test did not show heterogeneity between independent SNPs applied to the IVW estimates (Cochran’s Q = 10.21, *p* = 0.512) (Table 2, Figure 2D).

### 3.2. Reverse MR

To verify the existence of reverse causality, we performed a reverse MR analysis, which treated AS and adiponectin separately as exposure and outcome. After setting a genome-wide significance threshold of *p* < 5 × 10^−8^ and removing LD, four SNPs were identified as IVs of AS for reverse MR analysis, and more detailed information on the four SNPs is shown in Table 3. The F-statistics of these SNPs were all >10.

**Figure 3:**
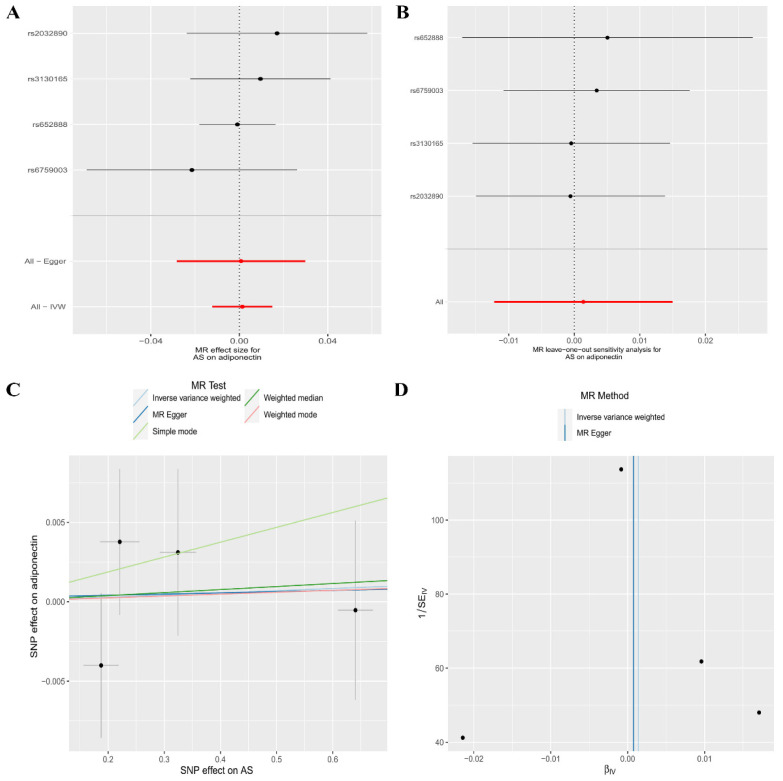
The association between the risk of ankylosing spondylitis and circulating adiponectin levels is presented in (**A**) a forest plot, (**B**) a leave-one-out sensitivity analysis, (**C**) a scatter plot, and (**D**) a funnel plot.

In reverse MR analysis, the random-effects IVW (OR, 1.00; 95% CI, 0.99–1.02, *p *= 0.842), MR–Egger regression (OR, 1.00; 95% CI, 0.97–1.03, *p* = 0.964), Weighted median (OR, 1.00; 95% CI, 0.99–1.02, *p* = 0.802), Weighted mode (OR, 1.00; 95% CI, 0.99–1.02, *p*  = 0.890), and Simple mode (OR, 1.01; 95% CI, 0.99–1.03, *p* = 0.474) methods all showed that there was insufficient evidence to support a genetic causal relationship between the risk of AS and CA levels (Table 4, Figure 3A). The Leave-one-out sensitivity analysis showed that our results were not biased by a single SNP (Figure 3B). Horizontal pleiotropy or heterogeneity was not observed in the MR–Egger regression (intercept = −0.000, *p* = 0.966) (Table 4, Figure 3C) or Cochran’s Q test (Cochran’s Q = 1.77, *p =* 0.621) (Table 4, Figure 3D), respectively.

## 4. Discussion

AS is an immune-mediated inflammatory arthritis that typically affects the sacroiliac and spinal joints, resulting in severe chronic pain and disability. It is frequently associated with multiple complications such as iritis, osteoporosis, and compression fractures of the spine, as well as cardiovascular disease, imposing a severe burden on the patients [35]. The etiology of AS is complex and cannot be fully explained by a single factor, but genetic factors have been a major focus of interest. As for genetic factors, the human leukocyte antigen (HLA) system is thought to have a strong association with AS. HLA-B27 is a protein located on the surface of the major histocompatibility complex (MHC)-I and is encoded by the *MHC B* gene on chromosome 6 [10]. An older study showed that it was responsible for 20% of the genetic susceptibility to AS [36]. Braun et al. reported that the risk of developing AS in HLA-B27-positive individuals was up to 5–7% [37]. Some studies have shown that patients who are HLA-B27-positive tend to exhibit worse clinical presentations and higher disease activity [38,39]. Moreover, HLA-B27 has been observed to accelerate new bone production in patients with AS [40]. In addition to HLA-B27, certain non-HLA-B27 also show a specific susceptibility to AS. A study aimed at investigating the association of HLA class I and II alleles with AS found that HLA-B60 was positively associated with AS [41]. Surprisingly, HLA-B60 was found to increase disease susceptibility 3–6-fold in HLA-B27-negative AS patients in a study based on Taiwanese AS patients [42]. Furthermore, HLA-B7, HLA-B16, HLA-B35, HLA-B38, and HLA-B39 are also relevant to HLA-B27-negative AS of different races [10]. Although HLA systems show increased susceptibility to AS, this trend is not independent. Multiple factors (immunological, microbiological, and endocrinological) may interact with the HLA system to increase susceptibility. A previous study showed that AS patients with HLA-B27-positive had increased expression of killer-immunoglobulin-like receptors in T cells and produced higher levels of interleukin-17A [43]. The gut microbiota is a complex and large homeostatic system of trillions of bacteria, and changes in its composition are associated with autoimmune diseases [44]. In a germ-free environment, transgenic HLA-B27 rats did not develop features of AS, however, after the introduction of bacteria, they showed a significant reversal [45,46]. This seems to suggest that the shared efforts of the gut microbial environment and HLA-B27 increase the genetic susceptibility to AS. Due to gender differences in AS, endocrine studies in AS are mainly based on sex hormone levels [10,47]. Moreover, in recent years, research on other endocrine hormones (including adiponectin) and AS has also been extensive [18,47,48].

The most abundant adipokine, adiponectin, has attracted a wide range of scholarly attention [49]. It can improve systemic energy homeostasis by promoting insulin sensitization and exerting a powerful protective effect against various physiological events by inhibiting cell death and suppressing inflammation [50]. Adiponectin is highly heritable, with an estimated heritability between 30 and 80% [26]. A previous study found significantly higher CA levels in AS patients with combined metabolic syndrome [51]. A study found significantly elevated CA levels in patients with AS compared with healthy controls [17]. We speculate that CA levels may also increase susceptibility to AS through genetic effects. Therefore, we used a two-sample MR study to explore the relationship between CA levels and AS at the genetic level, with potential value for further research on the etiology of AS.

Our MR results suggest that there is no causal genetic correlation between CA levels and the risk of AS. Meanwhile, there is limited supporting evidence to prove the causal impact of genetically predicted AS on CA levels. Currently, the evidence of CA levels in relation to AS is contradictory. Hartl et al. discovered that high molecular weight adiponectin levels were inversely associated with radiographic progression of the spine in AS [52]. Consistently, lower baseline adiponectin serum levels were also observed in AS patients with radiographic spinal progression after 2 years [52]. In contrast, a case-control study by Derdemezis et al. revealed that CA levels in patients with AS were notably higher than those in controls [17]. A previous study reported that adiponectin played a proinflammatory role in SpA [18]. These findings are inconsistent with our results in this study. Certainly, numerous studies support our present findings. For example, a study investigating the relationship between serum adipokines, clinical parameters, and radiographic progression in patients with AS showed that CA levels were not associated with disease activity or spinal radiographic progression [53]. A comparative study observed that total serum adiponectin levels were not correlated with the pathogenesis of AS [54]. Furthermore, numerous previous observational studies did not indicate significant differences in CA levels between patients with AS and controls [55,56]. A 2 year longitudinal study also reported similar results [57]. These results imply that CA levels do not have a causal relationship with the risk of AS.

The primary strength of our study is that we used a two-sample MR design, which diminishes the bias resulting from confounders and reverses causality. In addition, we manually removed SNPs associated with confounders to best satisfy the MR assumptions. Finally, all participants in our exposure-outcome GWAS dataset belonged to European ancestry, which avoided the bias caused by ethnic stratification. However, our study had several limitations. First, our data are derived from public databases, and further subgroup analyses cannot be performed to investigate the associations of specific factors (e.g., age and sex). Second, there may be a slight sample overlap in our study, which can generate the bias to some extent. Finally, the GWAS summary level data for CA we used was sampled from serum, and although adiponectin is predominantly distributed in plasma [14,58,59], a minor proportion was also present in synovial fluid, so the results of this study only focus on adiponectin levels in serum and ignore adiponectin in synovial fluid.

## 5. Conclusions

In summary, our MR study demonstrated that CA levels were not causally associated with the risk of AS. Furthermore, there is scarce evidence for the causal impact of the genetically predicted risk of AS on CA levels. Large-scale MR studies and clinical trials are required to validate our findings.

## Figures and Tables

**Table 1:** Detailed statistics of selected instrumental variables for circulating adiponectin levels and their relationships with AS.

SNP	MAF	Adiponectin			AS			R^2^	F-Statistic
		β	SE	*p*-Value	β	SE	*p*-Value		
rs12051272	0.009	−0.277	0.018	1.00 × 10^−200^	0.004	0.055	0.945	0.0014	55
rs1597466	0.092	−0.044	0.008	1.89 × 10^−08^	−0.108	0.062	0.082	0.0003	13
rs17366568	0.908	−0.154	0.009	1.00 × 10^−200^	0.009	0.044	0.844	0.0040	158
rs2062632	0.686	−0.055	0.006	2.52 × 10^−19^	0.008	0.036	0.823	0.0013	51
rs2927324	0.475	0.032	0.005	1.29 × 10^−11^	−0.005	0.031	0.878	0.0005	20
rs2980879	0.375	0.030	0.005	1.08 × 10^−08^	−0.025	0.034	0.459	0.0004	17
rs601339	0.150	0.039	0.006	3.87 × 10^−11^	−0.089	0.041	0.029	0.0004	15
rs6810075	0.633	−0.066	0.005	1.00 × 10^−200^	−0.026	0.030	0.394	0.0020	82
rs7615090	0.883	−0.058	0.008	2.81 × 10^−11^	0.011	0.043	0.791	0.0006	28
rs7955516	0.442	0.026	0.005	2.43 × 10^−08^	0.027	0.033	0.407	0.0007	14
rs7964945	0.808	0.037	0.006	2.61 × 10^−08^	0.020	0.040	0.617	0.0003	17
rs8042532	0.992	−0.340	0.055	2.86 × 10^−9^	−0.004	0.077	0.961	0.0004	76

SNP, single nucleotide polymorphism; MAF, minor allele frequency; SE, standard error; AS, ankylosing spondylitis.

**Table 2:** The results of a Mendelian randomization analysis that looked at the relationship between circulating adiponectin levels and the risk of ankylosing spondylitis.

Exposure	Method	SNP	OR	95% CI	*p*-Value	Heterogeneity *p*-Value (Cochran’s Q Statistic)	MR–Egger Intercept (*p*-Value)
Adiponectin	MR–Egger	12	0.98	0.70–1.36	0.901		0.003 (0.843)
	Weighted median	12	0.99	0.75–1.31	0.945		
	Inverse variance weighted	12	1.00	0.79–1.27	0.984	0.512 (10.21)	
	Simple mode	12	0.91	0.60–1.38	0.653		
	Weighted mode	12	0.98	0.75–1.28	0.890		

**Table 3:** Detailed statistics of selected instrumental variables for ankylosing spondylitis and their relationship with circulating adiponectin levels.

SNP	MAF	AS			Adiponectin			R^2^	F-Statistic
		β	SE	*p*-Value	β	SE	*p*-Value		
rs2032890	0.269	−0.221	0.035	2.97 × 10^−10^	−0.004	0.005	0.426	0.0191	4481
rs3130165	0.248	0.324	0.032	1.90 × 10^−23^	0.003	0.005	0.566	0.0392	9378
rs652888	0.219	0.640	0.031	7.00 × 10^−94^	−0.001	0.006	0.926	0.1404	37,516
rs6759003	0.664	−0.188	0.031	1.74 × 10^−9^	0.004	0.005	0.392	0.0157	3663

**Table 4:** The results of a Mendelian randomization analysis of the relationship between the risk of ankylosing spondylitis and circulating adiponectin levels.

Exposure	Method	SNP	OR	95% CI	*p*-Value	Heterogeneity *p*-Value (Cochran’s Q Statistic)	MR–Egger Intercept (*p*-Value)
AS	MR–Egger	4	1.00	0.97–1.03	0.964		0.000 (0.966)
	Weighted median	4	1.00	0.99–1.02	0.802		
	Inverse variance weighted	4	1.00	0.99–1.02	0.842	0.621 (1.77)	
	Simple mode	4	1.01	0.99–1.03	0.474		
	Weighted mode	4	1.00	0.99–1.02	0.890		

## Data Availability

The datasets supporting this study are available from IEU OpenGWAS (GWAS ID: ieu-a-1, https://gwas.mrcieu.ac.uk/ (accessed on 10 May 2020)) and the FinnGen consortium (https://www.finngen.fi/ (accessed on 9 June 2022)).
